# A Novel Framework for Understanding the Pattern Identification of Traditional Asian Medicine From the Machine Learning Perspective

**DOI:** 10.3389/fmed.2021.763533

**Published:** 2022-02-03

**Authors:** Hyojin Bae, Sanghun Lee, Choong-yeol Lee, Chang-Eop Kim

**Affiliations:** ^1^Department of Physiology, Gachon University College of Korean Medicine, Seongnam, South Korea; ^2^Korean Medicine Data Division, Korea Institute of Oriental Medicine, Daejeon, South Korea; ^3^Department of Korean Convergence Medical Science, University of Science and Technology, Daejeon, South Korea

**Keywords:** pattern identification, machine learning, dimensionality reduction, diagnostic system, traditional Asian medicine, traditional Chinese medicine, syndrome differentiation

## Abstract

Pattern identification (PI), a unique diagnostic system of traditional Asian medicine, is the process of inferring the pathological nature or location of lesions based on observed symptoms. Despite its critical role in theory and practice, the information processing principles underlying PI systems are generally unclear. We present a novel framework for comprehending the PI system from a machine learning perspective. After a brief introduction to the dimensionality of the data, we propose that the PI system can be modeled as a dimensionality reduction process and discuss analytical issues that can be addressed using our framework. Our framework promotes a new approach in understanding the underlying mechanisms of the PI process with strong mathematical tools, thereby enriching the explanatory theories of traditional Asian medicine.

## Introduction

Pattern identification (PI), a distinctive diagnostic system found in traditional Asian medicine (TAM), is a clinical reasoning process that uses the signs and symptoms of patients to identify diagnostic patterns (1). These patterns convey information about the nature of the disease or the location of lesions and serve as a guide for treatment selection (2) (e.g., drain for a “excess” pattern and tonify for a “deficient” pattern). Notably, patterns in TAM are pragmatic concepts that are widely accepted as a useful treatment target rather than actual pathogens or objectively measurable states (3). It can be said that PI is a strategy chosen to make diagnostic decisions based on naked sense observations and to determine corresponding treatments. Despite their centrality in theory and practice, the information processing principles of PI have remained relatively superficial. Additionally, abstract descriptions make it difficult to objectively describe the PI process, resulting in a low level of consistency between practitioners (4–6).

In recent years, approaches based on machine learning (ML) have demonstrated remarkable performance in a variety of tasks, including image classification, speech processing, and natural language processing, all of which are difficult to solve using knowledge-based approaches (7). Interestingly, this success has spawned approaches in systems neuroscience that use ML to study how the brain works (8–11). The strategy is to use ML algorithms as a computational model of the brain and to benchmark this model in order to gain a better understanding of how the brain represents, learns, and flexibly processes high-dimensional information.

Inspired by the idea that ML models can help capture critical aspects of the brain's computation, we present a novel framework for explaining how information is processed in the PI system and why it is effective. Within our framework, we model the PI system as a dimensionality-reduction algorithm and propose several research questions. By leveraging ML's framework, we can adopt powerful mathematical tools, broaden the scope of inquiry, and enrich explanatory theory in TAM.

## Manuscript Formatting

### A Brief Introduction to Dimensionality Reduction

In this paper, we view the PI system through the lens of dimensionality reduction process, which reduces high-dimensional data to a low-dimensional representation. To that end, we'll discuss high-dimensional data and dimensionality reduction briefly. Rather than providing strict mathematical definitions, we will explain these concepts with examples to aid intuitive understanding.

**Figure 1:**
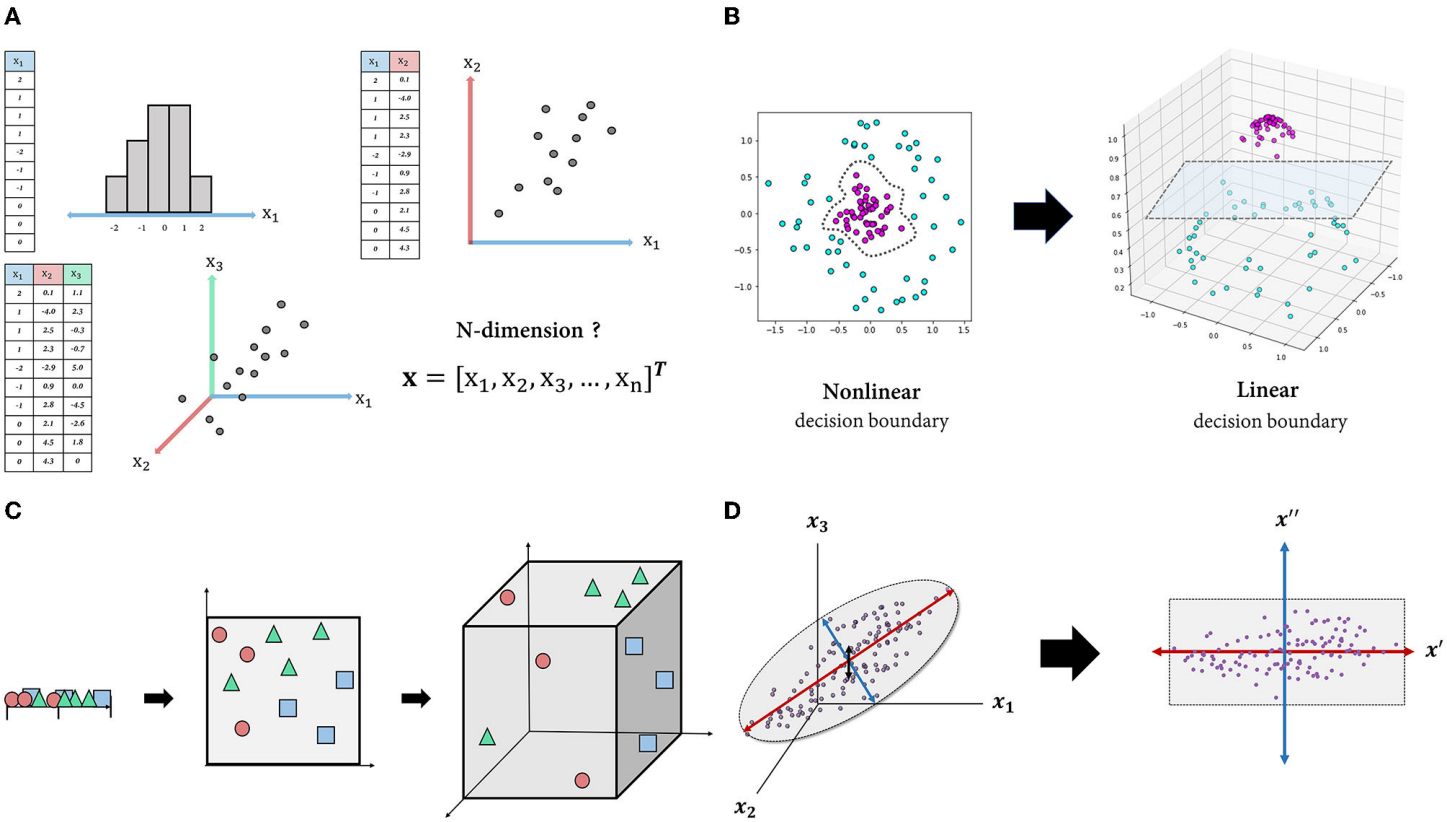
Schematic figures explaining the features of high-dimensional data. **(A)**: Intuitive understanding of multidimensional data. Each observation (row) in each table is described by one or more features (columns) and visualized as a point in one-, two- or three-dimensional space. This representation is easily extended to four-dimensional space or higher, but it cannot be visualized. **(B)**: By transforming the dataset into high-dimensional space, the data can be separated using a linear decision boundary. **(C)**: Curse of dimensionality. As the dimension of the space increases, the volume of the space expands exponentially, and the density of the space becomes increasingly sparse. **(D)**: Projection into an intrinsic-dimensional space. Data laid out in three-dimensional space can be approximated by a two-dimensional plane composed of newly discovered axes that account for majority of the data variability.

The dimensionality of data is defined as the number of features (attributes) that describe the observations in data (Figure 1A) (assuming that the number of rows (observations) exceeds the number of columns (features/attributes) and the data matrix is full-rank, which is easily satisfied in noisy, real-world datasets). A larger number of features leads to a more detailed representation of the observation (i.e., high representational power) (12). Additionally, when compared to low-dimensional space, high-dimensional space makes data classification easier (13). For instance, while classification in a low-dimensional space requires non-linear and complex decision boundaries, data can be made linearly separable by adding additional dimensions (axes) (Figure 1B).

However, high-dimensional space does not come without drawbacks. Due to the fact that more than four-dimensional space is beyond human cognition, high-dimensional data are unintuitive, making it difficult to interpret or derive insights. More importantly, as the input dimension increases, the classifier's performance on unseen data typically degrades rather than improves. A common explanation for this is the “curse of dimensionality” (14). As the dimension increases, the volume of space in which data are represented increases exponentially, to the point where available data become sparse (Figure 1C) (15). In this case, the model is likely to miss generalizable patterns in the data. One solution is to increase the size of the training data until the density is sufficient, while another is to reduce the dimensionality of the data, which is usually the more practical option (16).

Apart from these disadvantages of high-dimensional data, the typical motivation for dimensionality reduction is that the genuine dimension (i.e., degree of freedom) of the space may be significantly less than the number of features due to feature dependencies (17). That is, even if the dataset contains hundreds or even millions of features, the majority of variation may be explained by a handful of latent variables. There are numerous dimensionality-reduction algorithms, and which one to use depends on the nature of the data and the research objective. For instance, principal component analysis (PCA), one of the most widely used linear dimensionality-reduction techniques, seeks to identify orthogonal axes [i.e., principal components (PCs)] that best account for the variance of the data via a linear combination of existing axes (18). By projecting the data into a subspace of leading PCs, we can obtain a compact representation of the data, albeit with some information loss (Figure 1D). There are also non-linear techniques such as Isomap (19), t-stochastic neighbor embedding (20), uniform manifold approximation and projection (21) that capture non-linear relations between variables. Overall, the motivations for dimensionality reduction in dealing with high-dimensional data are as follows: first, high-dimensional data are unintuitive; second, they are prone to the curse of dimensionality; and third, a dataset's dimensionality may be artificially high.

### Modeling the PI as a Dimensionality-Reduction Process From the Symptom Space

**Figure 2:**
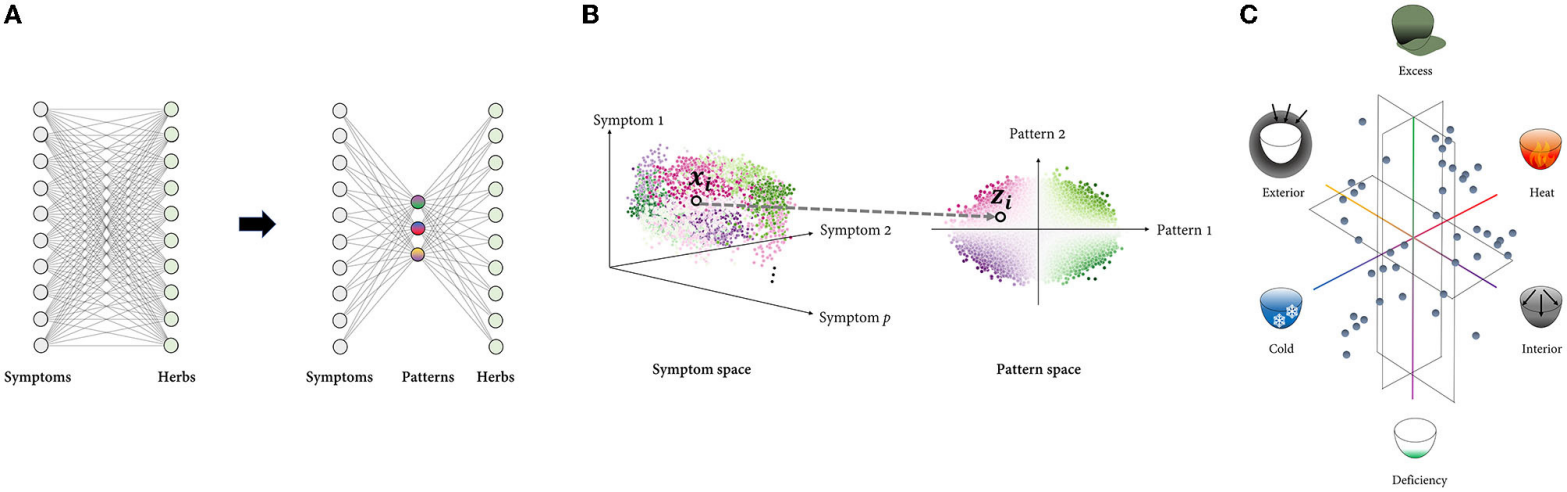
Framework modeling the PI with dimensionality reduction. **(A)**: Rather than mapping high-dimensional spaces directly, the number of cases can be reduced exponentially by first projecting the input space to low-dimensional space composed of multiple latent variables and then mapping it to the output space. **(B)**: Representing the data using a few underlying patterns reveals the intrinsic structure of the data, which is difficult to capture in a high-dimensional space where distinct factors of variations are highly entangled. Each point represents sample data, and the points denoted by a black circle represent the *ith* sample, which is represented in both the symptom space ($x_{i}\in R^{p}$) and the pattern space ($z_{i}\in R^{r}$). The points are color-coded according to the identified patterns. **(C)**: TAM's low-dimensional pattern space is constructed from metaphorical concepts that are embodied in everyday life. The pattern space in eight-principle PI, the most comprehensive type of PI, is visualized as an example. Six of eight principle patterns are composed of the exterior, interior, cold, heat, deficiency, and excess, while the other two are Yin and Yang, which are higher concepts that encompass the other six patterns. Six principle patterns are grouped in pairs of mutually opposing properties: exterior-interior, cold-heat, and deficiency and excess. These three pairs represent the extent to which external pathogens penetrate the body, the nature of the disease, and the relative superiority of the body's resistance to pathogenic factors and the pathogenic qi, respectively. These concepts' familiar and abstract characteristics enable robust inference of the pathological pattern from a myriad of symptom phenotypes.

One of the most distinctive characteristics of TAM in clinical practice is the use of patterns to identify and treat the patient. TAM physicians evaluate patient's clinical symptoms and signs and classify them according to specific pattern groups (4). The identified patterns provide basis for prescribing treatments including herbal formula (22). Each patient can be thought of as a point in a multidimensional symptom space, with each dimension corresponding to a distinct symptom. If the total number of symptoms is *p*, the patient is represented as a *p*-dimensional vector whose elements are the coordinate values on each symptom axis. Similarly, the herbal space can be defined in the same way, with each dimension representing an individual herb. If the total number of herbs is *q*, a herbal prescription (a mixture of herbs) is represented as a *q*-dimensional vector whose elements are the coordinate values for each herbal axis. Following that, treatment selection can be formulated as a mapping from the symptom space to the herbal space (To keep the discussion concise, treatment is limited to herbal prescriptions). From the doctor's perspective, there are several motivations to reduce the dimension of the input data to perform this task successfully. Assuming the symptom and herbal space have dimensions of *p* and *q*, respectively, the number of theoretically possible mappings is *q*^*p*^. Even if each *p* and *q* are on a tens-scale, they are already beyond the cognitive capacity of any single human memory. In this case, shrinking the input space's dimension can exponentially reduce the number of available alternatives (Figure 2A).

Additionally, there is frequently a high degree of correlation and redundancy among individual symptoms, limiting possible patterns of variation (e.g., fever may have a positive correlation with thirst and a negative correlation with a pale face). In other words, a small number of independent patterns can effectively describe the system's behavior, resulting in symptom data that may span only a constrained, low-dimensional subset of the entire space. In this case, the overall structure of the symptom data, which may not be visible at the individual symptom level, may be more important for treatment selection. Disentangling the data based on latent patterns may aid in revealing the data's intrinsic structure (Figure 2B).

Given this perspective, the PI system can be modeled as the process of representing high-dimensional symptom data in a low-dimensional space defined by a few latent patterns. Assuming that the patient records contain *p* symptom variables and are represented by *r* pattern variables, PI process can be described as follows: Given a set of *n* patient vectors $X={\left\{x_{i}\right\}}_{i=1}^{n}\;$[i.e., the *i*th training sample is a vector $x_{i}{=\left[x_{i1},x_{i2},\;\ldots ,\;x_{ip}\;\right]}^{T}$, where *x*_*ij*_ is *j*th feature of *i*th sample], the aim is to transform each vector $x_{i}\in R^{p}$ into a new vector $z_{i}\in R^{r}{=\left[z_{i1},z_{i2},\;\ldots ,\;z_{ir}\;\right]}^{T}$ where *r*≪*p*. The mapping function *f*:*X* ⊂*R*^*p*^→*Z*⊂*R*^*r*^can be estimated differently depending on the specific forms of the objective function.

Interestingly, human-inferred latent patterns may not always be the optimal solution in terms of information loss minimization, which is the primary goal of dimensionality-reduction algorithms such as PCA. This is because, given human expert's inductive reasoning, reduced representations must not only deliver compact information but also be cognitively efficient. Indeed, the patterns in TAM, such as heat, cold, deficiency, and excess, are primarily intuitive and metaphorical concepts that are embodied in daily life (Figure 2C). Inferring patterns from experiences or observations of the physical world and explaining the physiological and pathological phenomena of the human body in terms of these conceptual patterns are key characteristics of TAM theory (23). While this approach may appear crude and ideological in comparison to pathogen-based diagnosis, it provides an intuitive foundation for inductive reasoning (24, 25).

### Research Questions in PI Systems That Can Be Addressed Using Mathematical Metrics Developed in ML

In this section, we raise several research questions that can be addressed by utilizing our novel framework that models the PI system in terms of the ML perspective. In particular, we focus on the topics for specifying the dimensionality reduction properties of the PI system.

#### Is PI a Linear or Non-linear Process?

Dimensionality-reduction algorithms can be classified mathematically as linear or non-linear, which is critical for implementation. Linear techniques such as PCA, multidimensional scaling, and factor analysis are widely used in a variety of fields. They employ straightforward linear algebraic techniques that are easy to implement and provide clear geometric interpretations (26). In the real world, however, data may form a highly non-linear manifold. Low-dimensional embeddings obtained via methods assuming a linear submanifold may be unsatisfactory in this case (27).

Whether to use a linear or non-linear technique should be determined by the nature of the data being analyzed, as well as the nature of the problem being solved. The PI process should compress the symptom space while retaining the information required for treatment selection, but its linear or non-linear nature has not been investigated. For instance, the probability of being identified as a particular pattern can increase supra-linearly when a particular symptom pair appears concurrently, whereas the probability may be negligible in the absence of a single symptom.

Numerous techniques exist for quantifying non-linearity in operations (28–30). Quantifying non-linearity may allow for the assessment of the adequacy of currently developed tools supporting clinical PI. For example, a questionnaire based on linear regression may be ineffective for a disease in which significant non-linear associations exist between symptoms and patterns.

#### How and to What Extent the PI Abstracts Information

The core characteristic of human intelligence is to learn from small samples to deal with previously unknown situations, which are often linked with the critical challenges raised in ML (31). For the brain to learn efficiently within its limited resources, it is necessary to draw general conclusions from individual experiences rather than memorize them all (32). Abstraction and hierarchical information processing are critical capabilities that contribute to the human brain's remarkable capacity for generalization (7, 33).

Given that PI is the process of representing patients' clinical symptoms as metaphorical patterns, it is fundamentally an abstraction process. Abstract representations may aid physicians in robustly inferring pathological patterns from a wide variety of symptom combinations, thereby simplifying patient classification. It is critical to investigate how and at what level abstractions are made and how they contribute to patient classification and/or treatment selection in order to gain a better understanding of information processing in PI.

Obtaining abstract (high-level) representations while ignoring irrelevant details is also critical in artificial intelligence (AI). Deep neural networks, in particular, such as convolutional neural networks and autoencoders are thought to learn abstract representations, and abstraction in representations can be quantified in various ways, for example, the degree of dichotomy or the capacity for generalization (34–38). Similarly, abstractions in PI can be explicitly quantified using the PI model's representation. Whether or not TAM concepts with varying levels of abstraction are hierarchically encoded in the system, or whether the level of abstraction varies between different types of PI that employ distinct conceptual patterns, such as Qi and blood, viscera and bowels (zangfu), or the five phases, could be specific research topics. This would enable us to assess the appropriate level of abstraction as well as its advantages and disadvantages.

#### What Is the Objective Function of the PI System?

The objective function specifies how a model's performance/cost is calculated, and a model is trained to maximize or minimize it. In other words, the objective function represents the model's learning goal, which is a critical component that must be specified in ML practice along with the learning rule and the architecture (39). Similarly, we can consider the PI system's objective function. Investigating the objective function that led the development of TAM's clinical decision-making model into its current form will give insight on the information processing strategy of PI system.

We can start with a common objective function of ML to determine that of the PI system. In supervised learning, the most widely used objective function is as follows:


$$\hat{f}=\underset{f\epsilon ℋ}{arg\;min}[\frac{1}{n}\sum {}_{i=1}^{n}L(y_{i},\;f(x_{i}))\;+\lambda J(f)]$$

ℋ denotes the function space of *f*, and the function $\hat{f}$ is found by the minimization of the cost (inside the square bracket), which is composed of the loss function *L*(*y*_*i*_, *f*(*x*_*i*_)) and the regularization function *J*(*f*) with its associated regularization weight λ. *y*_*i*_ and *f*(*x*_*i*_) denotes the ground-truth treatment and model prediction for the *ith*-sample *x*_*i*_, respectively. To minimize the loss, the model should fit the training data as closely as possible. However, the complexity of the model is constrained by the penalty imposed by the regularization term. The strategy of having two conflicting components in the objective function enables the designer to consider a reasonable bias-variance trade-off (i.e., enhancing the model's reliability in the face of unseen data at the expense of greater bias) (40). In other words, the objective function formulation expresses explicitly which characteristics the system values and penalizes.

It will be important to investigate which type of performance or penalty should be assessed by the loss and regularization functions in order to induce the current PI system. For example, when describing the long-term evolution of a PI system, the regularization function may be used to constrain the agent's cognitive and/or computational load rather than to prevent overfitting (In the long run, variance shrinks because $\underset{n\to \infty }{\text{lim}}\sigma^{2}=0,\;$where σ^2^ denotes the model variance).

## Discussion

Earlier research on developing an AI-based diagnostic system for TAM was primarily focused on developing an expert system that makes use of expertise and ontology (41–45), whereas in recent years, a bottom-up approach that generates knowledge from the data has become more prevalent. The majority of recent PI studies utilizing ML have attempted to develop predictive models capable of reproducing a physician's diagnosis (46–48). While these studies explored the clinical applicability of ML algorithms based on their predictive performance, there were also studies examining the PI theory itself. One study validated TAM pattern types statistically by demonstrating that patient clusters in the data set correspond well to theoretical pattern types (49, 50), and another used a decision tree algorithm to extract a collection of symptoms indicative of a pattern in a particular disease (51) [For a more comprehensive and systematic review of the application of quantitative models in traditional medicine, see (52, 53)]. Our study is unique in that it presents a broader framework for explaining and analyzing PI system's information processing strategy from a ML perspective. Additionally, while we explained the PI process as dimensionality reduction, it is not exclusive to other ML algorithms such as clustering.

When dimensionality reduction is used to extract latent features, the process is comparable to that of theorization or modeling. Both involve deriving fundamental principles or patterns from massive and disordered data at the expense of detailed information. A model that fully describes all data samples is merely an enumeration of facts and is incapable of conveying generalized knowledge. Instead, we require a simple explanation to make sense of the data despite the presence of residuals that the model cannot account for. This aspect of dimensionality reduction is consistent with TAM's distinctive way of thinking, which seeks to interpret changes of the patient's symptoms and discomfort using abstract concepts that describe the dynamic nature of the micro-environment of the human body (54). By grasping the generalizable principles underlying individual observations, we can explain, predict, and manipulate the observed system's behavior beyond the scope of our experience.

According to cognitive psychology, humans frequently employ heuristic strategies that arrive at satisfactory solutions with a modest amount of computation to make decisions within their cognitive capacity and time constraints (55–57). Numerous models have been proposed to explain the strategies employed by the human brain, and dimensionality-reduction model in this paper is in line with such models. However, the constraint on computing resources in the dimensionality reduction of ML is not severe, resulting in differences between the human and machine computation. Additionally, it is expected that extensive feature selection will occur prior to dimensionality reduction in the actual PI process, based on cues such as the patient's chief complaint. This procedure would be based on the physician's prior knowledge, which correspond to the Bayesian prior. It is also noteworthy that reduced representations in PI systems must be interpretable because they are the product of conscious reasoning, unlike many ML algorithms, including PCA.

We combine the ingredients of systems neuroscience and ML to propose a conceptual framework for investigating the PI system, based on TAM domain knowledge. The introduction of a new perspective leads to the emergence of novel research questions and methodologies, opening a novel field of investigation. By implementing mathematical tools developed in ML, we will be able to verify a variety of hypotheses to which qualitative approaches have been applied primarily and contribute to the development of shareable explicit knowledge. This may help overcome one of TAM theory's primary flaws, namely that it is subjective and difficult to articulate. While this framework leaves room for elaboration, we believe it will serve as the foundation for developing interpretable AI for the medical domain.

## Data Availability Statement

The original contributions presented in the study are included in the article/supplementary material, further inquiries can be directed to the corresponding author.

## Author Contributions

C-EK, C-yL, and SL: conceptualization. HB and C-EK: investigation. HB: writing—original draft. C-EK and SL: writing—review and editing and funding acquisition. All authors contributed to the article and approved the submitted version.

## Funding

This work was supported by the National Research Foundation of Korea (NRF) grant funded by the Korean government (MSIT) (No. 2020R1F1A107317912) and the Collection of Clinical Big Data and Construction of Service Platform for Developing Korean Medicine Doctor with Artificial Intelligence research project (KSN2021110).

## Conflict of Interest

The authors declare that the research was conducted in the absence of any commercial or financial relationships that could be construed as a potential conflict of interest.

## Publisher's Note

All claims expressed in this article are solely those of the authors and do not necessarily represent those of their affiliated organizations, or those of the publisher, the editors and the reviewers. Any product that may be evaluated in this article, or claim that may be made by its manufacturer, is not guaranteed or endorsed by the publisher.
